# Fermentation quality, bacterial community, and predicted functional profiles in silage prepared with alfalfa, perennial ryegrass and their mixture in the karst region

**DOI:** 10.3389/fmicb.2022.1062515

**Published:** 2022-11-28

**Authors:** Xueying Fan, Zhiming Xie, Qiming Cheng, Maoya Li, Jianhua Long, Yao Lei, Yushan Jia, Yulian Chen, Chao Chen, Zhijun Wang

**Affiliations:** ^1^College of Animal Science, Guizhou University, Guiyang, China; ^2^College of Life Sciences, Baicheng Normal University, Jilin, China; ^3^Shibing County Agricultural and Rural Bureau, Shibing, China; ^4^College of Grassland, Resources and Environment, Key Laboratory of Forage Cultivation, Processing and High Efficient Utilization of Ministry of Agriculture, and Key Laboratory of Grassland Resources, Inner Mongolia Agricultural University, Ministry of Education, Hohhot, China

**Keywords:** alfalfa, perennial ryegrass, fermentation quality, bacterial community, predicted functional, karst region

## Abstract

There is little information regarding the dynamics of fermentation products and the bacterial community in silage prepared with alfalfa (MS), perennial ryegrass (LP), and their mixture in the karst region. In this study, we explored the effects of combining MS with LP in different ratios (100% MS, 70% MS + 30% LP, 50% MS + 50% LP, 30% MS + 70% LP and 100% LP; fresh matter basis) on silage chemical composition, fermentation quality, bacterial communities and predicted functions during the ensiling process. Each treatment was prepared in triplicate and stored at room temperature (22–25°C) for 7, 15, and 45 days. The dry matter (DM) and water-soluble carbohydrate content of the silages increased as the LP proportion in the mixed silage increased; at 45 days, the 70% MS + 30% LP, 50% MS + 50% LP and 30% MS + 70% LP silages contained higher (*p* < 0.05) CP content than the 100% MS and 100% LP silages. The 30% MS + 70% LP and 100% LP silages exhibited lower (*p* < 0.05) pH and higher (*p* < 0.05) LA content than the other silages; at 45 days, none of the silages contained PA or BA. As fermentation proceeded, the abundance of harmful (*Enterobacteriaceae* and *Sphingomonas*) and beneficial (*Lentilactobacillus*, *Lactiplantibacillus*, *Secundilactobacillus*, and *Levilactobacillus*) microorganisms decreased and increased, respectively, as the LP proportion in the mixed silage increased. The predicted functional distribution of microbial communities and metabolic pathways revealed that the 30% MS + 70% LP and 100% LP silages had a stronger capacity for fermentation and a weaker capacity for nitrate reduction than the other silages. Moreover, as the fermentation proceeded, the 30% MS + 70% LP and 100% LP treatments enhanced the functions of “Metabolism,” “Genetic information processing” and “Organismal systems” at level 1, the functions of “Amino acid metabolism” and “Nucleotide metabolism” at level 2, and the functions of “Metabolic pathways,” “Biosynthesis of secondary metabolites,” “Biosynthesis of antibiotics” and “Purine metabolism” at level 3. Thus, adding LP could improve the fermentation quality of MS silage by changing the composition and metabolic function of microbes; furthermore, ensiling 30% alfalfa with 70% ryegrass can produce high-quality silage in the karst region.

## Introduction

The region of Southwest China is a fragile area with grassland ecosystems and encompasses the most typical, complex, and impeccable development of karst worldwide (Ying et al., 2021). Due to the extreme environment, which involves rocky desertification, soil erosion, and soil depletion, herbivores often lack a food supply in winter and early spring; thus, the development of animal husbandry in this region is often restricted (Zhang et al., 2012; Yuan et al., 2021). Farmers have long stored forage as hay for supplementary feed in winter (Chen et al., 2017). However, dry matter (DM) and losses in nutritive value often occur during the haymaking stage. Furthermore, the karst area typically incurs high temperatures and precipitation at the same time, and compared to traditional hay, silage may be more suitable for the development of local animal husbandry (Zhang et al., 2015a).

Alfalfa (*Medicago sativa* L., MS), as a typical legume forage, is rich in crude protein and widely used as animal feed (Zhao et al., 2020). However, successfully ensiling MS is difficult due to its high buffering capacity, low water-soluble carbohydrate (WSC) content, and low epiphytic lactic acid bacteria (LAB) populations (Wang et al., 2020a). Ensiling MS in a mixture containing gramineous forage with high WSC content would be a good method of conservation (Wang et al., 2019). Numerous studies have shown that the fermentation quality of MS can be significantly improved by ensiling it in a mixture with gramineous forages (whole-plant corn or sweet sorghum; Zhang et al., 2015b; Wang et al., 2020a). Perennial ryegrass (*Lolium perenn* L., LP) is distributed widely in Asia, Europe, and northern Africa and exhibits several characteristics, including a long growing period, strong adaptability, and high forage yield (Su et al., 2021). LP can be preserved as ruminant feed through ensiling (Romero, 2010). However, the crude protein (CP) content in fresh LP and silage (96.1 g/kg; Dong et al., 2022) cannot meet the growth requirements of ruminants (NRC, 2007). Previous research demonstrated that to meet these requirements, legume forage with a high CP content can be mixed with low CP gramineous forages for ensiling (Zhang et al., 2015a). Therefore, ensiling MS with LP may be a good choice for improving the nutritional and fermentation quality of MS. However, few studies have investigated in detail the feasibility of mixing MS and LP for producing silage.

Ensiling is a fermentation process driven by microorganisms, especially LAB (Wang et al., 2022). In the fermentation process, lactic acid bacteria use WSC to produce lactic acid and other substances that lower the pH, thereby inhibiting other harmful microorganisms (such as *Enterobacter* and *Escherichia coli*; Zhao et al., 2020). Therefore, characterizing the microbial community is critical for further improving the quality of silage. To our knowledge, little information is available regarding the dynamics of fermentation products, bacterial communities and predicted functional profiles of silage prepared with MS, LP, and their mixture in the karst region. Therefore, this study was performed with the purpose of probing the effect of different ratios of MS and LP combinations on fermentation quality, bacterial communities and predicted functional profiles throughout the ensiling process. We hypothesized that (i) ensiling MS in a mixture with LP would enhance the nutritional and fermentation quality of silage, and (ii) increasing LP could improve the fermentation quality of MS silage by inhibiting harmful bacteria.

## Materials and methods

### Silage preparation

Alfalfa and perennial ryegrass were cultivated in Guanling County, Guiyang city, Guizhou Province. This region has a typical karst landscape, mainly with a humid mid-subtropical monsoon climate, with an average annual temperature of 16.2°C, an average annual precipitation of 1205.1–1656.8 mm, and an average altitude of 1,025 m. Fresh alfalfa (MS, Victoria varieties) was harvested at the early flowering stage, while perennial ryegrass (LP, Guicao NO.1 varieties) was harvested at the boot stage. Without wilting, each of the two forages was separately chopped to a length of 2–3 cm using a hand hay cutter. Approximately 300 g of chopped MS was mixed homogenously with LP, packed manually into polyethylene bags (25 cm × 30 cm), and then vacuum packed using a vacuum packing machine (SJ-400, Shanghai Precision Machinery Manufacturing Co. Ltd). The treatments were as follows: (1) 100% MS: 100% alfalfa+0% perennial ryegrass; (2) 70% MS + 30% LP: 70% alfalfa+30% perennial ryegrass; (3) 50% MS + 50% LP: 50% alfalfa+50% perennial ryegrass; (4) 30% MS + 70% LP: 30% alfalfa+70% perennial ryegrass; and (5) 100% LP: 0% alfalfa+100% perennial ryegrass. Three polyethylene bags of silage with the same treatment were stored at room temperature (22–25°C) and were opened in triplicate for each treatment after 7 and 45 days of ensiling to analyze microbial diversity, which was achieved by examining the chemical compositions and fermentation quality after 7, 15, and 45 days of ensiling.

### Analysis of microbial populations, chemical compositions, and fermentation quality

As performed by Cai et al. (1999), the plate count method was used to determine the microbial populations of fresh materials. Briefly, 10 g of MS or LP was homogenized with 90 ml autoclaved 0.85% sodium chloride solution, shaken at 120 rpm and 37°C for 2 h, and serially diluted from 10^−1^ to 10^−7^. The LAB, yeasts and coliform bacteria were counted by culture-based methods using deMan, Rogosa, and Sharpe (MRS) agar plates (GCM188, Land Bridge Technology Co., Ltd., Beijing, China), malt extract agar (CM173, Land Bridge Technology Co., Ltd., Beijing, China) and blue light broth agar (Nissur Ltd., Tokyo, Japan), respectively. The microbial number was enumerated in colony-forming units (cfu), converted to logarithmic form, and expressed on a fresh material (FM) basis.

Each sample was dried at 65°C, crushed, and sieved through a 1 mm sieve to estimate the dry matter (DM) content. The content of water-soluble carbohydrate (WSC) was determined by the anthrone method (Owens et al., 1999). In brief, a dried sample of 0.2 g was ground with a mill (1 mm screen), mixed with 10 ml of sterile distilled water in a glass tube and incubated at 100°C for 30 min. The filtrate was transferred to a volumetric flask, and diluted with sterile distilled water to 100 ml. One microliter of the solution was mixed well with 5 ml of anthrone-sulfuric acid solution (0.4 g anthrone+100 ml 88% sulfuric acid) and incubated at 100°C for 10 min. The absorbance of the mixture was measured at 620 nm. Glucose was used as a reference substance, and the standard curve was as follows: y = 0.117x + 0.008, R2 = 0.9996, where x is the concentration of the filtrate (mg/mL) and y is the absorbance value at 620 nm. The minimum detection concentration was 1.0 mg/ml. When the absorbance value was >1.2 and <0.2, the filtrate was diluted to achieve highly accurate determination. Crude protein (CP) was analyzed using a Kjeldahl analyzer (Kjeldahl 2300 Automatic analyzer, FOSS Analysis AB, Hoganas, Sweden) according to the methods of the official Society of Analytical Chemists (AOAC, 1990). Both the neutral detergent fiber (NDF) and acid detergent fiber (ADF) levels were analyzed using the methods of Van Soest et al. (1991).

Each 10 g silage sample was mixed homogeneously with 90 ml of sterile water and stored at 4°C for 6 h, followed by filtration through four layers of cheesecloth. The filtrates were finally stored in 50 ml centrifuge tubes at −20°C for subsequent analyzes. The filtrate was tested for organic acids, ammoniacal nitrogen (AN) and pH. The pH was measured by a pH metre. The AN content was determined by the sodium hypochlorite-phenol method (Broderick and Kang, 1980). The filtrate was centrifuged at 10000 r at 4°C for 10 min and analyzed with a 0.22 μm dialyzer. Lactic acid (LA), acetic acid (AA), propionic acid (PA) and butyric acid (BA) levels were determined by high-pressure liquid chromatography (HPLC; KC-811 column, Shodex; Shimadzu Co. Ltd., Tokyo, Japan; oven temperature, 50°C; mobile phase, 3 mmol/L perchlorate solution; flow rate, 1.0 ml/min; flame photometric detection wavelength, 210 nm; and sample size 5.0 μl; Jia et al., 2019).

### Bacterial community analysis

Microbial DNA was extracted from the silage sample according to the method described by Wang et al. (2020b). In brief, the preserved liquid for extracting DNA was centrifuged (rate, 12,000 × *g*; time, 10 min), and the pellet was collected and used to extract DNA by a Power Soil DNA Isolation Kit (MO BIO Laboratories) according to the manufacturer’s protocol. After purification, the DNA was diluted to 1 ng/ml using sterile water. All microbial DNA samples were immediately sent to Biomarker Technologies Corporation (Beijing, China) for PCR amplification and bioinformatic analysis. The 16S rDNA V3–V4 regions were amplified using a forward primer (50-ACTCCTACGGGAGGCAGCA-30) and reverse primer (50GGACTACHVGGGTWTCTAAT-30) combined with specific barcode sequences. FLASH software was applied to check the raw reads, and the high-quality sequences (scores >80) were saved based on the QIIME quality control process. The operational taxonomic units (OTUs) with 97% similarity were clustered by UPARSE pipeline software. Then, UCHIME software was used to identify and remove the chimeric sequences. The alpha diversity indices including the OTU, Ace, Chao, Shannon, Simpson and coverage indices, were determined using Mothur software. The metabolic potential of the bacterial community and the composition of functional genes were predicted by assigning 16S rRNA marker gene sequences to functional annotations of sequenced metagenomic sequences based on the Kyoto Encyclopedia of Genes and Genomes (KEGG) pathway Orthology (KO) classification, using Tax4Fun (version 0.3.1). The comparisons of the KEGG pathways were graphically presented *via* Graphpad Prism (version 8, IBM, Armonk, NY, United States).

### Statistical analysis

Data for the chemical composition, fermentation quality and the bacterial community were analyzed *via* two-way analysis of variance to evaluate the effects of ratios (R), ensiling period (P), and their interaction (R × P). All statistical analyses were performed using the general linear model procedure with SPSS 26 software (IBM Crop., Armonk, NY, United States). The data on the relative abundances of the KEGG pathways were subjected to one-way ANOVA. Tukey’s multiple comparison test was applied to analyse the statistical differences. Differences were considered as significant at *p* < 0.05. All the figures were created using Graphpad Prism (version 8, IBM, Armonk, NY, United States).

## Results and discussion

### Characteristics of raw materials

The chemical parameters and microbial populations of fresh samples are shown in Table 1. The DM contents of fresh MS and LP were 23.48% DM and 30.6% DM, respectively. The CP content of MS was 23.3% DM, which was similar to the result (24.6% DM) of Luo et al. (2021). The NDF (47.43% DM) and ADF (24.22% DM) contents of LP in our study were comparable to those reported by Li et al. (2019), who reported that the NDF and ADF contents of annual ryegrass were 48.39% DM and 28.16% DM, respectively. The NDF (50.49% DM) and ADF (26.41% DM) contents of MS were higher than those reported by Yuan et al. (2020), who reported that the NDF and ADF contents of MS were 44.00% DM and 20.80% DM, respectively. This shows that environmental conditions and varieties are the main factors affecting the nutritional quality of the material (Li et al., 2019). Silage quality depends on many factors, including the WSC content and epiphytic LAB count of raw material (Cheng et al., 2021). During ensiling, LAB convert WSC to organic acids, mainly LA, under anaerobic conditions, thereby lowering the pH to protect forage from undesirable microorganisms (Li et al., 2019). Therefore, ensiling is a LAB-driven fermentation process and the minimum requirement for the LAB count in raw materials is >10^5^ cfu/*g* FW (Wang et al., 2017). WSC acts as fermentation substrate, and the WSC content should be >50 g/kg of DM was shown to be crucial for ensuring acceptable fermentation quality (Seale et al., 1986). In our study, low numbers of attached LAB (< 10^5^ cfu/*g* FM) and high numbers of yeasts (10^3.52^ cfu/*g* FM) in fresh MS made it difficult to perform silage and necessitated the addition of LAB additives or high-sugar gram forages to improve silage quality (Zhang et al., 2017). Compared to MS, LP contained higher levels of LAB (10^4.97^ cfu/*g* FM) and WSC (19.59% DM) and lower levels of yeasts (10^2.31^ cfu/*g* FM). Therefore, we hypothesized that the addition of LP could promote the fermentation of MS silage.

**Table 1:** Chemical compositions and microbial numbers of alfalfa and perennial ryegrass prior to ensiling.

Item	Alfalfa	Perennial ryegrass
Dry matter (%FM)	23.48 ± 1.32	30.60 ± 1.21
Crude protein (%DM)	23.30 ± 1.04	8.22 ± 0.79
Neutral detergent fiber (% DM)	50.49 ± 0.83	47.43 ± 1.58
Acid detergent fiber (% DM)	26.41 ± 1.24	24.22 ± 1.37
Water soluble carbohydrate (% DM)	7.08 ± 0.62	19.59 ± 0.96
Lactic acid bacteria (Log cfu/g FM)	3.31 ± 0.22	4.97 ± 0.51
Yeasts (Log cfu/g FM)	3.52 ± 0.58	2.31 ± 0.63
Coliform bacteria (Log cfu/g FM)	<2.00	<2.00

FM, fresh matter; DM, dry matter; cfu, colony-forming unit.

### Dynamic changes In nutrient composition during ensiling

The chemical compositions of silages are shown in Table 2. The ratios (*p* < 0.01) and ensiling period (*p* < 0.01) significantly influenced the DM, CP and WSC contents of silages. The DM content is an important indicator in silage, as LAB need moisture for growth and reproduction (Oladosu et al., 2016). As fermentation proceeded, the DM content of silages gradually decreased. This reduction could be attributed to the metabolism of soluble substrates (e.g., WSC) by fermentative microbes (Oladosu et al., 2016). The DM content of silages increased as the proportion of LP in the mixed silage increased because the content of DM was higher in fresh LP than in fresh MS. In our study, as fermentation proceeded, the CP and WSC contents of silages gradually decreased because the nutrients were consumed by microorganisms (Dong et al., 2020). The CP content of silages at 7 and 15 days decreased as the proportion of LP in the mixed silage increased because the content of CP was lower in fresh LP than in fresh MS. Interestingly, at 45 days of ensiling, the CP content of MS and LP mixed silages was higher (*p* < 0.05) than that of MS or LP silage alone, which indicated that ensiling MS with LP may reduce the loss of CP. This may be because adding LP increased the abundance of beneficial microorganisms (such as LAB) in alfalfa silage, thereby inhibiting the degradation of CP by harmful microorganisms.

**Table 2:** Dynamic changes in nutrients in mixed silage of perennial ryegrass and alfalfa.

Items	Ratios (R)	Ensiling period (P)			SEM	*p* value		
		Day 7	Day 15	Day 45		R	P	R × P
DM (%FM)	100% MS	19.89Ab	16.51 Bd	16.31 Bd	0.307	<0.001	<0.001	0.101
	70% MS + 30% LP	21.09Ab	17.29 Bd	17.18Bc				
	50% MS + 50% LP	22.53Ab	18.24Bc	17.65Bc				
	30% MS + 70% LP	24.21Ab	19.96Bb	19.30Cb				
	100% LP	30.47Aa	21.57Ba	21.09Ba				
CP (%DM)	100% MS	22.35Aa	21.48Ba	16.17Bb	0.265	<0.001	<0.001	0.068
	70% MS + 30% LP	21.04Aa	19.17ABa	18.16Ba				
	50% MS + 50% LP	20.56Aa	17.20Bb	18.17Ba				
	30% MS + 70% LP	17.38b	16.72b	17.08a				
	100% LP	7.99c	7.41c	6.65b				
WSC (%DM)	100% MS	2.47A	2.19ABb	2.00Bab	0.101	<0.001	<0.001	<0.001
	70% MS + 30% LP	3.38A	2.17Bb	2.24Ba				
	50% MS + 50% LP	3.08A	2.53ABb	1.86Bab				
	30% MS + 70% LP	3.42A	2.70Ab	1.69Bb				
	100% LP	3.22B	4.52Aa	1.85Cab				

^A-C^Means of ensiling periods within a row with different superscripts differ on the same ratio treatment (*p* < 0.05); ^a−d^Means of ratios within a column with different superscripts differ on the same ensiling period (*p* < 0.05). DM, dry matter; FM, fresh matter; CP, crude protein; WSC, water-soluble carbohydrate; R, ratios; 100% MS, 100% alfalfa; 70% MS + 30% LP, 70% alfalfa + 30% perennial ryegrass; 50% MS + 50% LP, 50% alfalfa + 50% perennial ryegrass; 30% MS + 70% LP, 30% alfalfa + 70% perennial ryegrass; 100% LP, 100% perennial ryegrass; P, ensiling period; SEM, standard error of the mean; R × P, interaction of ratios and ensiling periods.

### Dynamic change in fermentation characteristics during ensiling

**Figure 1:**
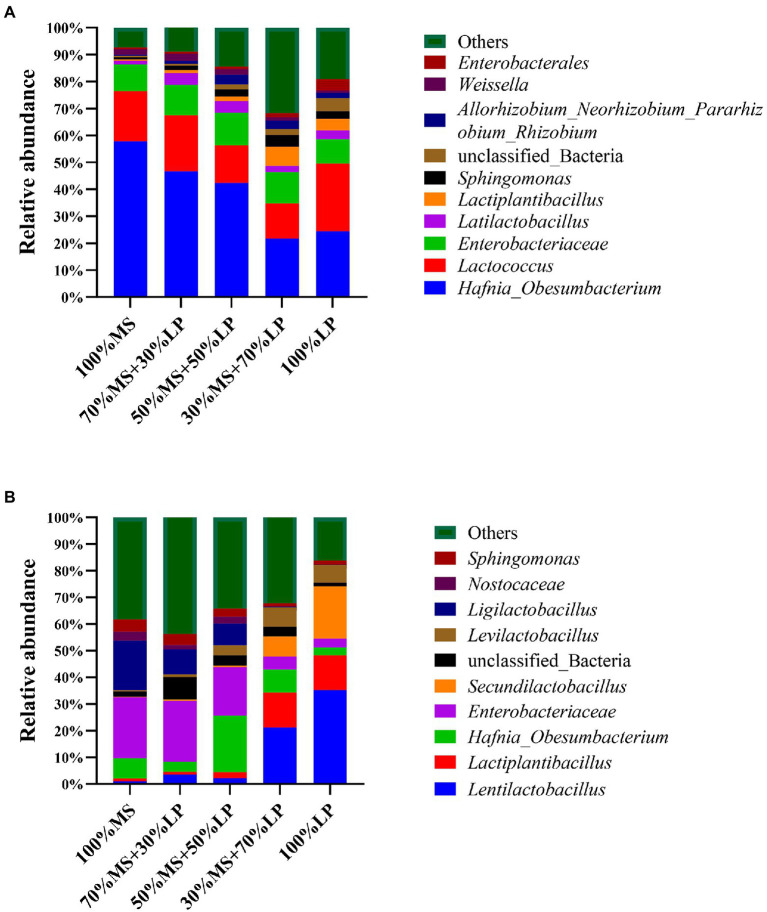
The relative abundance of bacterial communities at the genus level of alfalfa (MS) ensiled with perennial ryegrass (LP) silage for 7 **(A)** and 45 **(B)** days. 100% MS, 100% alfalfa; 70 MS + 30% LP, 70% alfalfa+30% perennial ryegrass; 50% MS + 50% LP, 50% alfalfa+50% perennial ryegrass; 30% MS + 70% LP, 30% alfalfa+70% perennial ryegrass; 100% LP, 100% perennial ryegrass.

The fermentation characteristics of the silages are shown in Table 3. The ratios (*p* < 0.01), ensiling period (*p* < 0.05) and their interactions (*p* < 0.01) significantly influenced the pH, LA and PA contents of silages. The key to limiting the growth of harmful microorganisms (e.g., enterobacteria or clostridia) is achieving quick early acidification (Pahlow et al., 2003). In our study, during 7–45 days of ensiling, the pH value of alfalfa silage (100% MS) first decreased and then increased slowly, which was consistent with the trend observed by Wang et al. (2020a). At 45 days of ensiling, the pH value of alfalfa silage was 5.82, which was higher than the study result (4.69) obtained by Wang et al. (2020a). This may be because the LAB count (10^3.31^ cfu/*g* FM) of fresh MS in our study was lower than that (10^6.69^ cfu/*g* FM) of Wang et al. (2020a). As expected, the fermentation quality of alfalfa silage was very poor, mainly manifested in high pH (5.82) and AN (5.50% DM) values and low LA content (0.68% DM). The pH value of silages decreased as the proportion of LP in the mixed silage increased, which was consistent with the result of Zhang et al. (2015c), who found that adding sweet sorghum could reduce the pH of alfalfa silage. This is due to the high WSC content of fresh LP, which provides a fermentation substrate for LAB growth and helps to reduce pH and increase LA content during the ensiling process (McDonald et al., 2002). At the early and late stages of ensiling (7 and 45 days, respectively), the LA content of silages increased as the proportion of LP in the mixed silage increased, which was consistent with the result of Dong et al. (2020), who reported that more organic acids, especially LA, could be generated by LAB when high WSC content forages were added to the silage. It is noteworthy that in the mixed silages, the LA content (8.37% DM) of the 30% MS + 70% LP treatment was significantly higher (*p* < 0.05) than that of the other treatments, while the pH value (4.63) was significantly lower (*p* < 0.05). This indicated that 30% MS + 70% LP treatment could better promote the growth of beneficial bacteria (e.g., LAB) and inhibit the growth of harmful bacteria. This result was confirmed by our microbial results (Figure 1B), in which the abundance of LAB (*Lentilactobacillus* and *Lactiplantibacillus*) in the 30% MS + 70% LP treatment was higher than that in the other treatments, while the abundance of *Enterobacteriaceae* was lower than that in the other treatments. It is well known that the AA, PA and BA contents of silage are undesirable (McDonald et al., 1991). The majority of AA is produced when heterofermentative LAB, propionibacteria, and enterobacteria interact with carbohydrates (McDonald et al., 1991). Propionibacterial activity partially contributes to the synthesis of silage PA (Woolford, 1975). The hallmark of a clostridial fermentation is that a significant amount of BA is generated in the silage (Pahlow et al., 2003). In our study, at 45 days of ensiling, the AA content was low in all silages, and PA and BA were not detected. The CP content was easily degraded and caused AN to accumulate during ensiling. As fermentation proceeded, the AN content of mixed silage in the late ensiling stage was lower than that in the early ensiling stage. This may be due to the dominance of LAB in the late ensiling stage, which inhibited the growth of AN-producing bacteria (Li et al., 2021). The AN content of silages decreased as the proportion of LP in the mixed silage increased in our study. This indicates that adding LP with a high sugar content can reduce the CP hydrolysis of MS silage (Wang et al., 2020a). At 45 days of ensiling, the AN content (1.48% DM) of the 30% MS + 70% LP treatment was significantly lower (*p* < 0.05) than that of the other treatments. In general, adding LP could improve the fermentation quality of MS silage, among which the silage quality of 30% MS + 70% LP treatment was the best; this improvement was mainly observed in a low pH (4.63), low AN content (1.48% DM) and high LA content (8.37% DM), and no PA or BA were detected.

**Table 3:** Dynamic changes in the fermentation quality of perennial ryegrass and alfalfa mixed silage.

Items	Ratios (R)	Ensiling period (P)			SEM	*p* value		
		Day 7	Day 15	Day 45		R	P	R × P
pH	100% MS	6.19Aa	5.80Ba	5.82Bab	0.037	<0.001	<0.001	0.001
	70% MS + 30% LP	5.92Aab	5.51Bb	5.94Aa				
	50% MS + 50% LP	5.67b	5.28c	5.62b				
	30% MS + 70% LP	4.86c	4.87d	4.63c				
	100%LP	4.46d	4.47e	4.34d				
LA (%DM)	100%MS	2.62Ac	2.36Ab	0.68Bc	0.394	<0.001	0.004	<0.001
	70% MS + 30% LP	3.60Ab	2.97Ab	0.43Bc				
	50% MS + 50% LP	5.53ABab	1.50Bb	2.24Bc				
	30% MS + 70% LP	6.21Aab	2.97Bb	8.37Ab				
	100% LP	8.31ABa	6.78Ba	12.14Aa				
AA (%DM)	100% MS	1.96Bb	2.80Aab	2.48AB	0.196	0.02	0.293	0.07
	70% MS + 30% LP	2.26b	3.15a	2.31				
	50% MS + 50% LP	2.45b	3.22a	2.12				
	30% MS + 70% LP	1.74ab	1.22b	1.77				
	100% LP	1.16Bb	1.30ABb	1.44A				
PA (%DM)	100% MS	0.07	0.15	ND	-	-	-	-
	70% MS + 30% LP	0.16	ND	ND				
	50% MS + 50% LP	0.17	ND	ND				
	30% MS + 70% LP	0.24	0.21	ND				
	100% LP	0.28	0.07	ND				
BA (%DM)	100% MS	ND	ND	ND	-	-	-	-
	70% MS + 30% LP	ND	ND	ND				
	50% MS + 50% LP	ND	ND	ND				
	30% MS + 70% LP	ND	ND	ND				
	100% LP	ND	ND	ND				
AN (%DM)	100% MS	6.94a	6.14a	5.50a	0.380	<0.001	0.294	0.799
	70% MS + 30% LP	4.87b	4.07a	3.48a				
	50% MS + 50% LP	3.67c	3.27b	2.85b				
	30% MS + 70% LP	3.13d	2.04c	1.48c				
	100% LP	2.98d	2.53c	2.18b				

^A-B^Means of ensiling periods within a row with different superscripts differ on the same ratio treatment (*p* < 0.05); ^a−d^ Means of ratios within a column with different superscripts differ on the same ensiling period (*p* < 0.05). DM, dry matter; FM, fresh matter; LA, lactic acid; AA, acetic acid; PA, propionic acid; BA, butyric acid; AN, ammonia nitrogen; R, ratios; 100% MS, 100% alfalfa; 70% MS + 30% LP, 70% alfalfa + 30% perennial ryegrass; 50% MS + 50% LP, 50% alfalfa + 50% perennial ryegrass; 30% MS + 70% LP, 30% alfalfa + 70% perennial ryegrass; 100% LP, 100% perennial ryegrass; P, ensiling period; SEM, standard error of the mean; R × P, interaction of ratios and ensiling periods.

### Bacterial diversity and abundance during ensiling

The bacterial diversity of the silage mixtures is shown in Table 4. The inconsistent pattern of changes in alpha diversities (mainly the OTU, Ace, Chao, Shannon and Simpson indices) of silages with different percentages of LP added indicated that adding LP significantly affected the species and abundances of microbes in MS silage. The average good coverage for all samples was over 0.99, indicating that the sequencing process was adequate to characterize the dynamic change in the bacterial community (Cheng et al., 2022). In our study, the values of the OTU, Ace, Chao, Shannon and Simpson indices in 30% MS + 70% LP and 100% LP silages decreased sharply as the fermentation proceeded, which was in accordance with the results of Dong et al. (2020). This may be due to the presence of fresh material in an aerobic and neutral environment, which favors the reproduction of epiphytic aerobic microorganisms. In the early ensiling stage (7 days), the internal environment in silage had not yet reached a strictly anaerobic state. However, the acidic and anaerobic environments in silages were greatly developed after 45 days of ensiling, leading to a significant decrease in the bacterial diversity. This also suggests that the acidic and anaerobic environmental stresses had a significant effect on the reproduction and growth of microorganisms in silages, and this effect was more pronounced in the late ensiling stage.

**Table 4:** Diversity and richness of the bacterial microbiota of alfalfa ensiled with perennial ryegrass.

Treatments		OTUs	Ace	Chao	Shannon	Simpson	Coverage
100% MS							
	Day 7	396	458	477	0.605	2.354	0.999
	Day 45	420	456	467	0.879	4.696	0.999
70% MS + 30% LP							
	Day 7	424	481	481	0.717	2.942	0.999
	Day 45	429	455	462	0.890	4.777	0.999
50% MS + 50% LP							
	Day 7	465	512	519	0.770	3.574	0.999
	Day 45	403	443	464	0.844	4.294	0.999
30% MS + 70% LP							
	Day 7	508	544	548	0.906	4.977	0.999
	Day 45	434	467	485	0.899	4.493	0.999
100% LP							
	Day 7	456	489	491	0.848	4.162	0.999
	Day 45	437	470	477	0.763	3.450	0.999

100% MS, 100% alfalfa; 70% MS + 30% LP, 70% alfalfa + 30% perennial ryegrass; 50% MS + 50% LP, 50% alfalfa + 50% perennial ryegrass; 30% MS + 70% LP, 30% alfalfa + 70% perennial ryegrass; 100% LP, 100% perennial ryegrass; OTUs, operational taxonomic units.

Changes in the bacterial community composition during the fermentation process in silage mixtures at the genus level are shown in Figure 1A (7 days) and Figure 1B (45 days). In the early ensiling stage (7 days, Figure 1A), *Hafnia_Obesumbacterium* (21.64–57.75%) was the predominant genus in all silages, and the abundance of *Enterobacteriaceae* (9.10–12.21%) was relatively high. The abundance of harmful bacteria (*Hafnia_Obesumbacterium* and *Enterobacteriaceae*) in the 30% MS + 70% LP and 100% LP treatments was lower than that in the other treatments. According to a prior study, the presence of *Enterobacter* in silage increases the pH, which encourages the growth of other aerobic microorganisms (Zhang et al., 2015c). This was the main reason why the pH of the 30% MS + 70% LP and 100% LP treatments (< 4.9) was significantly lower than that of the other treatments (>5.6; Table 3). In the early ensiling stage, the main LAB genus was *Lactococcus* (13.11–25.21%), while the abundance of *Latilactobacillus* and *Lactiplantibacillus* was low. This was consistent with the study by Cai et al. (1998), who believed that lactic acid-producing cocci initiate lactic fermentation at the early ensiling stage, while lactic acid-rod is critical for pH reduction at the late ensiling stage. This also confirmed that the predominant LAB genus in the late ensiling stage was lactic acid-rod (including *Lentilactobacillus*, *Ligilactobacillus*, *Lactiplantibacillus*, *Secundilactobacillus*, and *Levilactobacillus*), and by this time, the *Lactococcus* had disappeared completely (Figure 1B).

In the late ensiling stage (45 days, Figure 1B), *Enterobacteriaceae* (21.13–35.26%) was the predominant genus in the 100% MS, 70% MS + 30% LP and 50% MS + 50% LP silages, and *Lentilactobacillus* (21.13–35.26%), *Lactiplantibacillus* (12.98–13.185%) and *Secundilactobacillus* (7.54–19.66%) were the predominant genera in the 30% MS + 70% LP and 100% LP silages. We speculated that the high number of *Enterobacteriaceae* and low number of LAB genera in 100% MS, 70% MS + 30% LP and 50% MS + 50% LP silages were responsible for the poor fermentation quality (Table 3). It is noteworthy that the abundance of *Ligilactobacillus* (18.59%) in alfalfa silage alone was higher than in other treatments, but this did not improve its fermentation quality. This result indicates that ensiling is a microbial-driven process, and the synergistic effect of various microorganisms is the key to improving the fermentation quality of silage (Ni et al., 2018). In our study, the abundance of harmful microorganisms (including *Enterobacteriaceae* and *Sphingomonas*) decreased and the abundance of beneficial microorganisms (including *Lentilactobacillus*, *Lactiplantibacillus*, *Secundilactobacillus*, and *Levilactobacillus*) increased as the proportion of LP in the mixed silage increased. These results indicated that adding LP could improve the fermentation quality of alfalfa silage by changing the microbial composition, and the 30% MS + 70% LP and 100% LP treatments provided the best effect.

**Figure 2:**
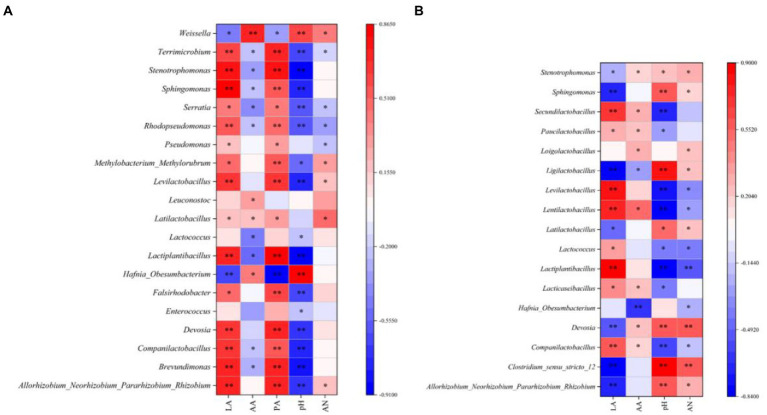
Spearman correlations between the silage fermentation parameters and the top 20 genera at 7 days **(A)** and 45 days **(B)**. LA, lactic acid; AA, acetic acid; PA, propionic acid; AN, ammonia nitrogen; *p values* are shown as * 0.01 < *p* ≤ 0.05, ** 0.001 < *p* ≤ 0.01; 100% MS, 100% alfalfa; 70% MS + 30% LP, 70% alfalfa+30% perennial ryegrass; 50% MS + 50% LP, 50% alfalfa+50% perennial ryegrass; 30% MS + 70% LP, 30% alfalfa+70% perennial ryegrass; 100% LP, 100% perennial ryegrass.

In the present study, a Spearman correlation between fermentation parameters and the top 15 genera during silage ensiling was established, as illustrated in Figure 2A (7 days) and 2b (45 days). Abundant microorganisms helped to enhance the fermentation quality of silage, and numerous metabolites impacted the microbial process (Li et al., 2022a). In our study, as fermentation proceeded, *Levilactobacillus* and *Lactiplantibacillus* were positively correlated with the LA concentration but negatively correlated with the pH value, which further confirmed that these two genera exhibited strong resistance to acids; in addition, these genera played a crucial role in the decreasing pH during the late stage of ensiling (Yuan et al., 2019). In the early ensiling stage (7 days), *Leuconostoc* was positively correlated with the AA concentration and disappeared at the late stage of ensiling. This is because *Leuconostoc* is a heteromorphic LAB that can produce AA, is less acid-resistant than other bacteria and is replaced by other LAB (e.g., *Loigolactobacillus*, *Lentilactobacillus*, *Paucilactobacillus*, *Secundilactobacillus*, *Ligilactobacillus*, and *Lacticaseibacillus*) as the silage pH continues to decrease (Cai et al., 1998). In our study, some harmful microorganisms (including *Falsirhodobacter*, *Pseudomonas*, *Rhodopseudomonas*, *Serratia*, and *Terrimicrobium*) were positively correlated with the PA concentration, suggesting that these genera might be mainly responsible for the high level of PA in silages at the early ensiling stage.

### Predicted functions of the microbial community in silages

**Figure 3:**
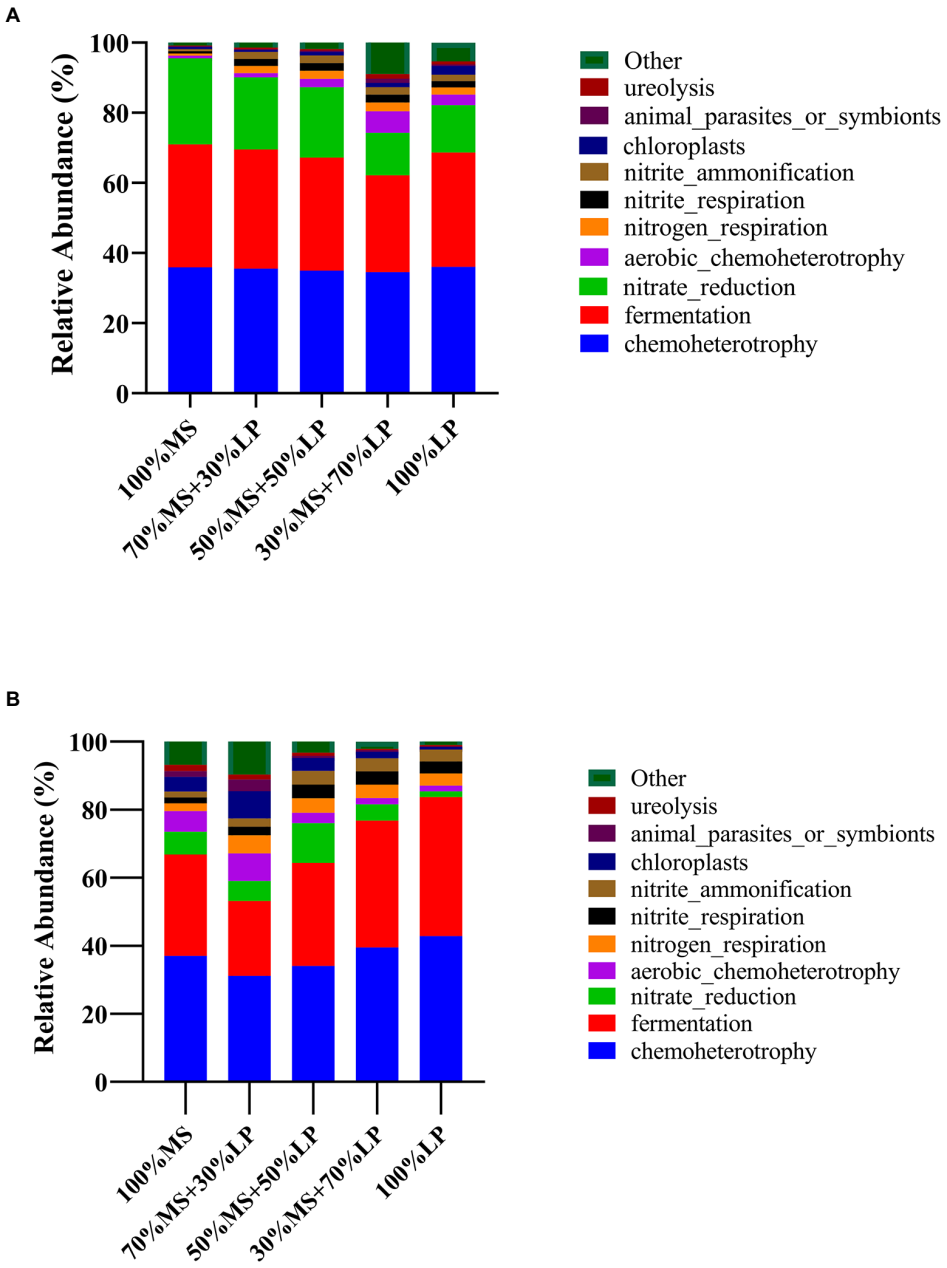
Dynamics of predicted microbial functional profiles of the relative abundances of the top 10 functions in alfalfa (MS) ensiled with perennial ryegrass (LP) silage for 7 **(A)** and 45 **(B)** days analyzed by Tax4Fun. 100% MS, 100% alfalfa; 70% MS + 30% LP, 70% alfalfa+30% perennial ryegrass; 50% MS + 50% LP, 50% alfalfa+50% perennial ryegrass; 30% MS + 70% LP, 30% alfalfa+70% perennial ryegrass; 100% LP, 100% perennial ryegrass.

Predicting the function profiles and metabolic pathways of the microbial community contributes to evaluating the influence of microbes on silage quality (Wang et al., 2020b). The Kyoto Encyclopedia of Genes and Genomes (KEGG) is a bioinformatics resource that is used to understand the function and utility of cells and organisms from a high level and genomic perspective (Wang et al., 2020b). Therefore, the effect of adding LP on the metabolic pathways of the microbial community in MS silage was determined using a Tax4Fun-based KEGG pathway. The results of functional prediction for the microbial community of silages during ensiling are shown in Figure 3. Chemoheterotrophy was the primary functional component of the microbial community in all silages, followed by fermentation, nitrate reduction, aerobic chemoheterotrophy and nitrogen respiration, which was similar to the results of Li et al. (2022b), who found that chemoheterotrophs were the primary functional components of the bacterial population in paper mulberry silage, followed by fermenters. In the early ensiling stage (7 days, Figure 3A), the functions of chemoheterotrophy (34.56–36.05%) and fermentation (27.57–35.02%) of all silages showed little change. Interestingly, 30% MS + 70% LP and 100% LP silages had a weaker capacity for nitrate reduction than other silages, and as the fermentation time proceeded, the nitrate reduction ability of the late ensiling stage (45 days) was weaker than that of the early ensiling stage (7 days). Previous studies have found that some bacteria reduce nitrate to nitrite, ammonia or nitrogen through the function of nitrate reduction, which was the main reason why the AN content of the 30% MS + 70% LP and 100% LP treatments was lower than that of the other treatments and the AN content gradually decreased as the fermentation time proceeded (Table 3; Xiang et al., 2022). In the late ensiling stage (7 days, Figure 3B), the 30% MS + 70% LP and 100% LP silages had a stronger capacity for fermentation than the other silages. This was because the 30% MS + 70% LP and 100% LP treatments showed increased abundances of LAB, such as *Lentilactobacillus*, *Lactiplantibacillus*, and *Secundilactobacillus*, and decreased abundances of *Enterobacteriaceae* (Figure 1B); the degree of fermentation was greater in silage (Li et al., 2022b). Furthermore, the 30% MS + 70% LP and 100% LP treatments had a weaker capacity for chloroplasts, animal parasites or symbionts and ureolysis than the other treatments. This may be because the abundance of beneficial microorganisms in the 30% MS + 70% LP and 100% LP treatments was higher than that in the other treatments, and the fermentation capacity was stronger, resulting in a low pH environment, which inhibited the activity of other harmful microorganisms (Ni et al., 2018).

**Figure 4:**
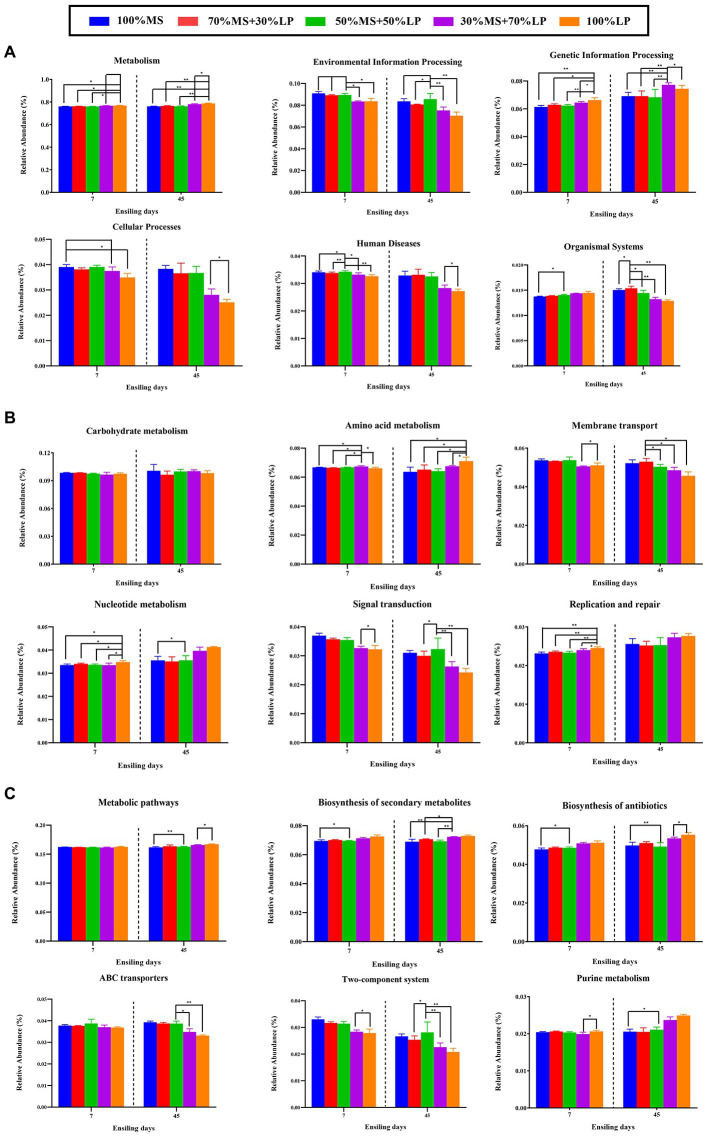
Bar graphs showing 16S rRNA gene-predicted functional profiles on the level 1 metabolic pathways **(A)**, level 2 metabolic pathways **(B)** and level 3 metabolic pathways **(C)** of silages for 45 days obtained with Tax4Fun. 100% MS, 100% alfalfa; 70% MS + 30% LP, 70% alfalfa+30% perennial ryegrass; 50% MS + 50% LP, 50% alfalfa+50% perennial ryegrass; 30% MS + 70% LP, 30% alfalfa+70% perennial ryegrass; 100% LP, 100% perennial ryegrass. ∗, 0.01 < *p* < 0.05; ∗∗, 0.001 < *p* < 0.01.

The results of the metabolic pathways of the microbial community of silages ensiling for 45 days are shown in Figure 4. As shown in Figure 4A, a total of six different metabolic pathways were observed in all the silages. The main predicted genes were assigned to metabolism, accounting for >76% in all the silages, followed by environmental information processing, genetic information processing and cellular processes, which is in accordance with a previous report (Hussain et al., 2021). After 7 days of ensiling, the 30% MS + 70% LP and 100% LP treatments had lower abundances of “Environmental information processing,” “Cellular processes” and “Human diseases” and higher abundances of “Metabolism,” “Genetic information processing” and “Organismal systems” than the other treatments. Based on the fermentation quality of the different treatments, the main reason for the better fermentation quality of the 30% MS + 70% LP and 100% LP treatments was the altered cellular characteristics, inhibition of the membrane transport and signal transduction of undesirable bacteria, and acceleration of the proliferation rate and metabolic level of beneficial bacteria (such as LAB strains; Wang et al., 2020b). After 60 days of ensiling, a low variation in metabolic pathways (except “Organismal systems”) at the first pathway level was found among the different treatments. This may be due to the fact that a stable internal environment had developed during the final stage of fermentation (Wang et al., 2020b). The carbohydrate metabolism mainly contained gluconeogenesis and glycolysis metabolism (Kanehisa and Goto, 2000). In our study (Figure 4B), as the fermentation proceeded, the changes in the abundance of “Carbohydrate metabolism” were not significantly different among all the treatments. This confirmed our results (Table 2), and there was little difference in WSC content among the treatments during fermentation. After 7 and 45 days of ensiling, the 30% MS + 70% LP and 100% LP treatments had lower abundances of “Membrane transport” and “Signal transduction” than the other treatments. This is consistent with the results of Keshri et al. (2018), who observed a higher abundance of transporters in the silage with poor fermentation quality, which may be related to the symbiotic relationship in the bacterial community. Furthermore, as the fermentation proceeded, the 30% MS + 70% LP and 100% LP treatments had higher abundances of “Nucleotide metabolism” and “Replication and repair” than the other treatments. According to Kilstrup et al. (2005), nucleotides may be used to synthesize and replicate RNA and DNA as well as serve as the primary source of energy for cellular processes. This demonstrated that LAB strains in the 30% MS + 70% LP and 100% LP silages multiplied rapidly during the early stage of the ensiling, which was in accordance with the highest abundances of LAB (including *Lentilactobacillus*, *Lactiplantibacillus*, *Secundilactobacillus*, and *Levilactobacillus*) in the 30% MS + 70% LP and 100% LP silages (Figure 1B). Amino acids, as essential substances in plants, are essential to promote primary metabolism and plant protein synthesis. In this study, as the fermentation proceeded, “amino acid metabolism” in the 30% MS + 70% LP and 100% LP treatments was promoted, while it was inhibited in the other treatments. Previous studies have found that the level of amino acid metabolism might reflect the ability of bacterial populations in silage to *de novo* synthesize amino acids (Keshri et al., 2018). Therefore, the observed dynamics of amino acid metabolism in silage may reflect the metabolism of the dominant populations throughout the ensiling process. The top 6 metabolic pathways at level 3 are shown in Figure 4C, and “Metabolic pathways” and “Biosynthesis of secondary metabolites” were the critical metabolic pathways at level 3. Interestingly, as the fermentation proceeded, the 30% MS + 70% LP and 100% LP treatments had higher abundances of “Metabolic pathways,” “Biosynthesis of secondary metabolites,” “Biosynthesis of antibiotics” and “Purine metabolism” and lower abundances of “ABC transporters” and “Two-component system” than the other treatments. This indicated that the 30% MS + 70% LP and 100% LP treatments could inhibit the transport of harmful bacteria in the membrane system by promoting the synthesis of metabolites in the silages for LAB strain propagation and reducing the pH of the silages (Wang et al., 2020b).

## Conclusion

In this study, we showed that the addition of perennial ryegrass with high WSC could affect the fermentation quality and microbial community of alfalfa silage by different metabolic pathways. Adding perennial ryegrass significantly improved the fermentation quality of alfalfa silage by increasing the abundance of beneficial microorganisms (e.g., *Lentilactobacillus*, *Lactiplantibacillus*, and *Secundilactobacillus*) and decreasing the abundance of harmful microorganisms (e.g., Enterobacteriaceae) in silages. By combining nutritional quality, fermentation quality and microbial community analyses, it was determined that ensiling 30% alfalfa with 70% ryegrass can result in high-quality silage in the karst region.

## Data availability statement

The datasets presented in this study can be found in online repositories. The names of the repository/repositories and accession number(s) can be found below: NCBI project PRJNA778048 with accession number SRP344966.

## Author contributions

ZX, QC, YJ, CC, and ZW designed the experiments and revised the manuscript. ML, JL, XF, and YL performed the experiments. QC, XF, and YC wrote the manuscript. ZX, ZW, and QC carried out the data analysis. All authors contributed to the article and approved the submitted version.

## Funding

This work was supported by the National Key Research and Development Subject (2021YFD1300302), Guizhou University Introduced Talents Scientific Research Project [Guida Renji Hezi (2020)71], Guizhou University Cultivation Project [Guida Renji Hezi (2020)9], and Jilin Province Science and Technology Development Plan Project (20190303075SF).

## Conflict of interest

The authors declare that the research was conducted in the absence of any commercial or financial relationships that could be construed as a potential conflict of interest.

## Publisher’s note

All claims expressed in this article are solely those of the authors and do not necessarily represent those of their affiliated organizations, or those of the publisher, the editors and the reviewers. Any product that may be evaluated in this article, or claim that may be made by its manufacturer, is not guaranteed or endorsed by the publisher.
